# Pain in Laboratory Animals: The Ethical and Regulatory Imperatives

**DOI:** 10.1371/journal.pone.0021578

**Published:** 2011-09-07

**Authors:** Larry Carbone

**Affiliations:** Laboratory Animal Resource Center, University of California San Francisco, San Francisco, California, United States of America; University of South Alabama, United States of America

The paradoxical goal of much of animal-based biomedical research is to model severe human injuries and illnesses without causing severe pain or distress to the animals. Current public policy in most countries calls for treatment or prevention of laboratory animal pain whenever possible. However, the “whenever possible” provision allows for some intentional infliction of untreated pain in laboratory animals when doing otherwise would be expected to disrupt the experiment. Permission to withhold painkillers when their use would interfere with the experiment is codified in public policy, as in the United States Department of Agriculture's 1971 designation of “Category E” painful procedures. Determining which experiments may permissibly cause pain and distress in laboratory animals, and deciding how that pain may be minimized or managed, requires clear ethical reasoning as well as the best available knowledge of animal biology and behavior. This article explores some of the common reasons why some laboratory animals may not receive pain medicines, and discusses some proposals for increasing use of pain medications for them. The policy focus is on the American system, though the general themes apply equally to other countries' laboratory animal welfare rules.

## Introduction

### Can it ever be ethical to leave pain untreated in laboratory animals?

Despite advances in the development of alternatives to using animals in research, scientists still often cite a need to use live animals in experiments. Once that need has been confirmed by funding agencies and/or the local IACUC (Institutional Animal Care and Use Committee) or other ethics committee, but before the research plans are finalized, the potential for animal pain and distress must be assessed, and plans laid to minimize animal suffering.

Regulatory concern for the pain of laboratory animals is not new. In 1970, the United States Congress updated the Animal Welfare Act (AWA) in calling for “adequate veterinary care, including the appropriate use of anesthetic, analgesic or tranquilizing drugs, when such use would be proper in the opinion of the attending veterinarian” [1]. This policy is echoed in a 2009 report of the National Academy of Sciences on the pain of laboratory animals, expanding pain management to a more generalized obligation than simply an aspect of the veterinarian's duties: “Laboratory animals need not experience substantial or ongoing pain and . . . prevention and alleviation of pain is an ethical imperative” [2]. The eighth edition of the *Guide for the Care and Use of Laboratory Animals* similarly states that “institutions are expected to provide oversight of all research animals and ensure that pain and distress are minimized” [3].

The ethical principle underlying laboratory animal welfare policy is that causing pain and distress to sentient animals is permissible, but requires strong justification [4]. It is a nuanced norm: causing pain is not categorically prohibited; it is allowed only with the justification that a valuable scientific experiment requires that animal pain be left untreated. Taking laboratory animal pain seriously does not equate to demonstrating a zero tolerance for animal pain.

Without question, present public policy allows humans to cause laboratory animals unalleviated pain. The AWA, the *Guide for the Care and Use of Laboratory Animals*, and current Public Health Service policy all allow for the conduct of what are often called “Category E” studies – experiments in which animals are expected to undergo significant pain or distress that will be left untreated because treatments for pain would be expected to interfere with the experiment [3], [5], [6]. One example among many would be studies of new painkillers for arthritis, in which a control cohort of animals are left untreated, while the experimental groups receive the test painkillers (which themselves may prove not to give pain relief).

But how are we to determine when to allow these experiments, or what limits to set on them? To move the mandate for “pain management when possible” from platitude to real-world guidance on difficult decisions requires carefully engaging the question of Category E experiments.

In this paper we assume that animal research will continue into the foreseeable future and that if regulations remain largely as they are, there will be times when scientists, veterinarians, and the local IACUC will agree that some pain will be left untreated. We discuss how that commonly happens, how things have changed over the years since the publication of Russell and Burch's seminal work, and close with some suggestions on how the process might be improved.

## Analysis

### Current American policy and practice

In 1959, William Russell and Rex Burch laid out their framework for identifying and then reducing “inhumanity” in animal experimentation. They pointed out that animal suffering may be a direct result of experimentation (e.g., some studies of pain may require pain to be inflicted), or it may be “contingent,” incidental to the study but not required for it (e.g., studies of advanced cancer may focus on finding cures, but maintaining animals with advanced cancer may mean that animals are in pain – almost by accident – that is in no way required for the experiment).

Clearly, the animals do not know whether the source of their pain is “direct” or “contingent,” and, fortuitously, Russell and Burch's suggestions apply to both types. Their proposal was to pursue, when possible, the overlapping “3Rs” of alternatives in and to the use of laboratory animals: *refine* animal procedures so that they cause less pain or distress; *reduce* the numbers of sentient animals on projects that can cause pain or distress; and, finally, *replace* sentient animals with nonanimals or non-sentient animals [7]. The 3Rs have been embraced in myriad policies, regulations, and articles, and they standardize our metric of progress toward the improved well-being of laboratory animals.

Current American policy and practice comprise two related norms: 1) causing animals significant pain and distress *must* be justified, and 2) causing animals significant pain and distress *can* be justified. In brief, a scientist can likely secure IACUC approval to cause serious pain, with few if any experimental procedures entirely beyond consideration. But the system is not *laissez-faire*: this approval can be gained only after strong justification and consideration of alternatives has been presented to the IACUC [8].

In 1985, the United States Interagency Research Animal Committee published its *Principles for the Utilization and Care of Vertebrate Animals Used in Testing, Research, and Training* (“the *Principles*”) [9]. The Public Health Service Policy on Humane Care and Use of Laboratory Animals and the AWA, the two main federal laws governing the care and use of laboratory animals, both hew closely to the *Principles* [6], [10].

The key precepts relevant to animal pain that are set forth in the *Principles*, as well as the two federal laws, include the following:

- Assume that what is painful to people is painful to animals;
- Avoid or minimize discomfort, distress, and pain when consistent with sound scientific practices; and
- Withhold tranquilizers, anesthesia, analgesia, or euthanasia only when scientifically necessary for only the necessary period of time.

Note that the *Principles* calls for scientists to minimize animal pain while at the same time allowing painful studies to be performed.

Note also, as has been discussed in detail by the present author (L.C.) elsewhere, the implicit principle that “pain counts; death doesn't” [11]. In brief, rather than death being treated as a harm to animals that must be minimized, death and killing instead exist in policy as the ultimate painkiller: *not* killing animals, at least, not killing animals in pain, is what requires justification. This principle is in fact crucial to the ethical justification of current practices in animal research. Killing, or euthanasia, is both a primary strategy for managing pain for many animals, especially those on chronic studies, and the fate of the overwhelming majority of laboratory animals, whether they are in sickness or in health.

Cancer pain is an exemplar. Virtually every human cancer is modeled in animals, and as in people, some cancers (such as oral and bone cancers) appear to be quite painful, even in their early stages. They can likewise be quite resistant to painkillers. In humans and companion animals, chronic cancer pain management includes progressively more aggressive opioid treatment with the most potent opioids (pure *mu* agonists such as fentanyl and morphine) [12]. Successful pain treatment can require an intravenous catheter for round-the-clock medication. This would be an extremely unlikely and challenging management strategy for rodents on cancer studies. Add to this that many pain drugs can have at least some effect on the progression of these cancers, and therefore might confound the research data [13], [14]. For these reasons, euthanasia is the main pain management strategy for such experiments.

Application of animal welfare policy is placed at the level of the institution, rather than on individuals in the institution. Whereas 1970 AWA provisions placed animal pain management within the provision of “adequate veterinary care” under the jurisdiction of the facility veterinarian, the 1985 amendment, still in force in 2011, shifted jurisdiction. While the researcher must consult with a veterinarian in planning potentially painful studies, it is now the IACUC (on which the veterinarian is one voting member) that reviews and approves or rejects a researcher's justifications for not treating animal pain. This decentralized decision-making is subject to some oversight – by the United States Department of Agriculture if the research involves the species they cover, by the Association for the Assessment and Accreditation of Laboratory Animal Care (AAALAC) in those facilities that voluntarily seek its accreditation, or by the National Institutes of Health's Office of Laboratory Animal Welfare (for institutions that receive Public Health Service funding).

Thus, current public policy clearly allows for harmful uses of laboratory animals. It nonetheless pushes scientists to take seriously Russell and Burch's “three Rs”: refinement, reduction, and replacement [7]. However, the “when scientifically necessary” clauses of current policy do not require that all procedures be fully refined such that laboratory animals will not suffer pain or distress, they do not call for reduction to zero of animal use in painful experiments, and they do not call for complete replacement of sentient animals with other models.

### Progress in finding alternatives in animal research

How can we measure progress toward a goal of no *unnecessary* animal pain, or how can we meet the far bigger challenge: *no* pain or suffering at all for laboratory animals?

In practice, in reviewing proposals to use animals, IACUCs consider what pain is likely to occur and what plans are laid out to prevent, minimize, or treat it. Although the deliberations of IACUCs are mostly internal affairs, they are publicly reflected in annual reports to the USDA. The most recent of these, labeled “Annual Reports of Enforcement,” are available online at the USDA's Animal Care Web site. USDA has been collecting these reports since 1971, and therefore could be a powerful resource for tracking progress over the years [15], [16].

Inspection of the USDA's on-line annual reports for 2002–2009 indicate that, very roughly, 7–9% of all animals used in research, teaching, or testing are reported in Category E, i.e., they are used on studies in which pain or distress are left untreated because painkillers would affect the data being collected. The pattern appears to be that the numbers are going down, from approximately 100,000 ten and more years ago to approximately 76,000 in 2008 and 2009 [17].

One might assess progress toward a goal of zero animal pain by studying these trends in the USDA reports, but several factors make this presently impossible. First, the reports cover only AWA-covered species, a tiny fraction of the animals used in research (the author of the present article estimates that the AWA covers less than 1% of research mammals, as laboratory-bred mice and rats are presently excluded from AWA coverage, including the annual reporting requirements). Available evidence does not allow us to know whether the (mostly larger) species covered by the AWA accurately reflect the fate of laboratory rats and mice. For example, if the vast majority of animal studies of cancer or pain are performed in small rodents, those entire areas of research are largely invisible to the AWA and its annual reports.

Further frustrating a longitudinal assessment of USDA's data are inconsistencies in reporting. For example, during some years, until USDA clarified that they should not do this, some facilities reported their numbers for rats and mice, while others did not. Far more significant is the fact that Category E standards have changed over the decades. Years ago, if an animal underwent surgery with a general anesthetic, followed by no postoperative pain medication, she/he might be reported in Category D (in which potentially painful procedures are treated with painkillers). This would in fact be keeping with the standard of care in veterinary practice of the day, in which post-operative analgesics were not standard for dog or cat spays or many other surgeries [18]. By today's standards, although the same anesthetics might be used, if there is no follow-on analgesic treatment, many institutions would place these animals in Category E.

This evolution of what counts as a Category E procedure may reflect changed mores about animal pain, but these changing mores have happened contemporaneously with changes in the available information. Methods of pain diagnosis continue to evolve, as do the available medications for treating pain.

The USDA data combine pain and distress, and so it is impossible to sort out those animals who experience distress without pain. For example, studies that induce fear, though with no pain inflicted, could be reported in Category E.

There is a threshold question in placing animals in Category E. Minor, short-duration pain (think of a flu vaccine) would not generally call for use of painkillers, and would not put an experiment in Category E if painkillers were not administered. The threshold for distress is provided by example. A low threshold of food deprivation—“Food and/or water deprivation or restriction beyond that necessary for normal presurgical preparation,” or about six to 10 hours—could put an experiment into Category E [19].

Thus, by today's standards for both pain and distress, the USDA Category E numbers from previous decades should be much higher than actually reported at the time. If the numbers of Category E animals are slowly decreasing, while the threshold for Category E has been lowered, that might well mean that the amount of significant pain in AWA-covered animals is in fact decreasing. As noted, we do not have good data to determine how the numbers reported for AWA-covered species reflect the fate of laboratory mice and rats,

Another source of data on trends in pain and pain management is examination of published experimental reports. In one such survey, Richardson and coworkers found relatively low use of animal analgesics being reported, but 4 years later a similar survey by Stokes and colleagues found an increase [14], [15]. The limitation of these reports, and of future follow-on studies faithfully using their methods, is determining whether painkillers were used and not reported, or not used at all.

To be clear, not every experiment is painful. Further, not every painful experience, at least in humans, is painful enough to warrant the use of painkillers. Thus, two important questions arise: 1) are animals getting enough pain management to keep them out of significant pain, and 2) if they are not, is their pain truly limited to what is scientifically necessary? Asking whether laboratory animals *should* get more pain medication requires looking at some facts about the management of animal pain and further probing values about the use of animals in laboratories.

### Facts and values in animal ethics

#### Case 1

Researcher G wants to model myocardial infarction (MI) in mice to explore whether a proposed treatment with muscle growth factors has promise for humans suffering an MI, or “heart attack.” An MI is surgically induced in mice by opening the chest and tying off a coronary artery so that a section of heart muscle loses its blood supply in a manner roughly comparable to the way that clots choke the blood supply to human heart muscle during a spontaneous MI. The question is: how much of which painkillers should these mice receive?

In cases like this it seems useful to identify the relevant facts (empirical claims) and values to reach a normative (“what ought we do”) conclusion or prescription (see box):

### Making a Decision: Facts + Values → Prescriptions

#### The facts

The values of respecting animal suffering underlie the public policy to treat animal pain whenever possible. This case study illustrates the application of that principle, and the need to clarify the facts, and to further refine the values.

For this study, we can list several questions of fact that need answers before we can prescribe a treatment regimen, including:

- Can mice feel pain?
- How much does chest surgery hurt the mouse?
- Is acute ischemia (a “heart attack”) painful in mice?
- What signs might mice exhibit when they are feeling pain?
- What analgesics can successfully treat the pain? At what dose and frequency?
- How will pain medications affect the heart data being collected?
- How will untreated pain affect the heart data being collected?
- How well do studies on mice model a human MI?
- What side effects on mouse health do the painkillers cause?

Some of the fact-questions have clear and simple answers, but often the answers are unknown (as when talking about the inner feelings of mice, especially of various genetically modified strains of mice). Or, the answers might best be expressed probabilistically (*x*% of mice will experience significant pain with this surgery; *y* % of mice will benefit from three times daily buprenorphine pain medicine), probabilities that may vary with mouse strain, skill of operator, or other factors [20].

#### Ethical decision-making: facts plus values

Answers to these fact-questions can help us decide how to proceed – if a mouse MI does not faithfully model the corresponding event in humans, there is no justification for the study; if mice do not feel pain from the procedure, there is no need to worry about painkillers. The facts alone are insufficient to answer the normative question. If mice feel pain, but one's values exclude moral concern for murine pain, then Dr. G may continue unencumbered. On the other hand, if mice feel pain, but there is no moral justification to cause others (here, mice) pain, then Dr. G cannot do the study.

In the simplest case, the painkillers have no [known] effect on the experimental data. Dr. G has an obligation to give her mice analgesics to the extent her veterinarian can recommend safe and effective medications. How much expense must she incur if the medicines are costly? How much inconvenience, if they require midnight re-dosing to get a mouse comfortably through the night? Humans have their own reasons not to self-medicate for every single pain. But in making these decisions for animals who have no liberty to avoid the experiment or to self-medicate, researchers must be vigilant in determining how much their own convenience, their own failure to see the animals' pain, or their failure to adequately research concerns about experimental outcomes can lead to undertreatment of animal pain.

Dr. G has no desire for the mice to be in pain, and she may not balk at the cost or inconvenience of pain drugs. She wants to see whether muscle growth factor works in a beating heart as it did in heart cells in her lab. But she also knows that the common classes of analgesic drugs – opioids like morphine and nonsteroidal anti-inflammatory drugs (NSAIDs) like ibuprofen – have effects throughout the body, and she fears their use will cloud her interpretation of the data. If the growth factor failed to work, could it be simply that the NSAID was to blame? Did she ruin her experiment in following her veterinarian's pain management recommendations?

Animal studies have an advantage over human studies in their ability to control so many extraneous sources of variability in the data. For many scientists (hence the number of Category E studies being done), experiments may seem ‘tainted’ when excess drugs, such as NSAIDs, are used. A new report from the National Academy of Sciences, however, puts this concern in a different perspective: “In studies where the use of certain analgesics appears to be contraindicated, investigators should be mindful that unwanted variables from pain-induced perturbation of homeostatic mechanisms can affect the animal model” [2]. In other words: do not eliminate painkillers as sources of unwanted variability without carefully assessing the effects of pain itself.

Less obvious, perhaps, are the effects that pain could have on efforts to model human disease. We know that human MIs are not caused by doing open-chest surgery, and we also know that a surgical MI in the mouse will release a host of inflammatory mediators, and these will lead to pain. Furthermore, the pain of a chest incision may make breathing more difficult, and the decreased ventilation could create various research artifacts. In addition, pain may make animals less likely to eat and drink – how might this affect the heart's response to the experimental growth factors in a metabolically challenged animal?

Saving a principle such as “treat animal pain when possible” from sitting in a frame on the wall as a hollow platitude requires *quantification*. The facts must be quantified: how much pain? How much of an effect of pain, or of painkillers, on data? And the values must be quantified: how much pain warrants how much cost, inconvenience, and what limits to pain treatment do various research projects merit? Importantly, normative decisions on how to proceed in the face of uncertain or probabilistic information must be made explicit.

To sum up, facts and values must be used together to make prescriptions, but the process is by no means straightforward. Different investigators, and different IACUCs, will differ on how they treat the prospect of pain in laboratory animals.

## Results and Discussion

### Moving forward: some suggestions

How should we move toward a goal of less and less laboratory animal pain? Following are some possibilities, some more realistic than others, and some more research friendly than others.

#### 1) Include fuller information in the literature

The USDA has stated its belief (with no evidence provided, however) “that the performance of a database search remains the most effective and efficient method for demonstrating compliance with the requirement to consider alternatives to painful/distressful procedures” [19]. That may one day be true, but for now it is hampered by the lack of animal-welfare-relevant information in too many research articles. For example, a recent literature review indicated that in 2006, less than 5% of mouse research articles and 27% of primate research articles reported on the implementation (or justification for non-use) of painkillers [21]. These results suggest that the USDA's assertion may be of little value to researchers.

This may and should change. The new “ARRIVE Guidelines” for publishing animal studies do recommend that details of anesthesia and analgesia be published in any animal studies [22]. Guidelines under development by the National Academy of Sciences will likely cover this as well [23]. Until the information is in the literature, however, a simple literature search, even with the term *alternatives* included, will not truly meet the informational needs of scientists, IACUCs, and veterinarians.

#### 2) Search better for information

Until the formal literature is far richer in detail on the management of animal pain in experiments, researchers and veterinarians need to look more broadly for information on alternatives. Various commercial search engines may pick up information missed by PubMed and the like, and list-serves for scientists and laboratory animal care specialists are useful for anecdotal information and experience. Cross-talk between the laboratory animal care community and the scientists they serve is essential.

#### 3) Generate better data

Refining animal research requires far more data on pain recognition in assorted species that we may find hard to “read,” such as mice, birds, frogs, and fish. Developing this knowledge, i.e., doing the pain research on these species, without causing the very pain we are still learning to read is a significant ethical challenge. We can add to this our need for more information on safe and effective pain medications at the right dose and frequency for these animals, and for data on how pain and painkillers both affect various research models. Guidelines on pain studies in animals are available to minimize, but not totally eliminate, the pain caused to the animals [24].

#### 4) Clarify what it means to “affect the model.”

As more is learned both about the far-reaching effects of various drugs throughout the body and the far-reaching effects of pain and distress on immune function, behavior, cancer biology, and more, clear thinking on how to balance these unwanted variables is needed. The simplistic approach is to look at the different outcomes (e.g., in cancer metastasis) of using an analgesic versus not using one in an experiment, and if any difference is found, to decide that the analgesic introduces extraneous variability and must be banished [25]. But if pain-treated and pain-untreated animals have different outcomes, is it not just as plausible that their pain is the source of artifact? Certainly, scientists should be absolutely clear about just what it is they are modeling before ruling out painkillers in their experiments. Working against this principle, alas, is the legitimate desire to tie ongoing work as closely as possible to that which has preceded it, to allow better comparison of findings. Staff turnover and changes in housing, animal genetics, and the availability of various medications are all potential sources of difference between prior work and future work, and against this backdrop, introducing the use of painkillers combined with good use of control subjects may become an acceptable refinement, even at the risk of diminished comparison with historical information. As noted above, and in the National Academy of Sciences publication, no researcher should rule out painkillers in her studies without having carefully investigated the effect of pain itself on her research model.

#### 5) Change the standard of care in use of analgesics

Like analgesics, anesthetics have wide-ranging and long-lasting effects on the animal as a research model. And yet, it is virtually unheard of to allow surgical procedures without anesthesia. Judicious use of concurrent control animals and standardization in the use of anesthetic can minimize the amount of variability and artifact that anesthetics may cause in an experiment. The rest of the anesthetic's effect must simply be accommodated in a world in which surgery without anesthesia is all but banned. Use of painkillers after surgery or for ongoing pain has not universally achieved that status in animal research, but one day it may well do so. Currently, researchers may justify withholding peri-operative analgesics out of concern for their effects on the model, and receive approval to do such Category E studies. An alternative standard would be to require *some* post-operative pain management, just as intra-operative anesthesia is now near-universally required, recognizing that some effect on the experiment is likely.

#### 6) Continue to develop ethical standards in IACUC review

The American system is decentralized, with authority vested in local IACUCs. On some issues, the IACUC may find little guidance, and different IACUCs may therefore develop quite different standards. As consensus emerges, or contentious issues are brought to the regulators' attention, guidance (or regulation) becomes more explicit. For example, there exists a tension between the “two R's” of reduction and refinement: is it better to use more animals with less pain or distress per animal (possibly a refinement), or to reduce numbers of animals by imposing more on each animal [26]? The 2011 *Guide* attempts to promote inter-institutional consistency and ethical clarity to this, in stating “reduction should not be a rationale for reusing an animal or animals that have already undergone experimental procedures especially if the well-being of the animals would be compromised” [3].

#### 7) Set limits on animal suffering by discipline

Presently, in theory at least, granting agencies disperse finite research funds to none but the best proposals. Society sets the limits, through donations to funding organizations, through government funding agencies, or through market-driven pharmaceuticals research. Disciplines or fields of inquiry compete amongst each other, uncommon non-life-threatening illnesses receiving much less funding than widespread serious illnesses. By contrast, IACUC approvals are in theory, unlimited. Would it be possible to reframe “permission to cause animal pain” as a finite resource that would be limited by discipline [27]? It is an intriguing concept, but presently unfeasible, in the present author's assessment, if only because we cannot measure cumulative animal pain in quanta that are nearly so clear as measuring research dollars. For now, the indirect way in which “pain per discipline” is limited is through limitations in funding.

#### 8) Pledge to end animal pain and distress

The Humane Society of the United States has an ongoing campaign urging colleges and universities to pledge to allow no severe unalleviated pain or distress in laboratory animals [28]. This pledge goes beyond federal policy that requires justifying untreated pain in Category E studies to actually banning it. There may be some word parsing, however: if Category E studies allow “more than minor or momentary” pain or distress, perhaps only a subset of these crosses the Humane Society's “severe” threshold. Even so, there may be some lines of inquiry, or, at least, some types of experiments, that would simply have to be set aside for this pledge to be honored. Animal studies of the mechanisms of the intense pain of advanced cancer, for instance, would seem to be off-limits, however beneficial for patients solving the problem of cancer pain would be. Full implementation of the pledge, banning what we would call “Category E-plus” research no matter the hoped-for benefits, would surely require a societal (and probably, regulatory) shift.

#### 9) Develop better reporting of “pain categories” in animal use

The USDA developed its system of reporting pain and distress in animals in 1971, with a goal of tracking progress toward full implementation of the 3 Rs [29]. Unclear definitions, shifting standards, and exclusion of the overwhelming majority of laboratory animals have limited the usefulness of these annual reports. A broader scale, better identifying studies presently on the D–E cusp, or establishing a new category of “E-plus” severe pain studies, applied to all research vertebrates, would improve this system.

### Conclusion

If present trends continue within laboratory animal science, the advent of new technologies will refine the use of animals in studies, reduce their numbers, and move us closer to large-scale replacement. Better recognition of pain and improved treatments should lead to less pain. Including fuller detail of animal pain management practices in the scientific literature will better disseminate information on “best practices” and elevate the standard of laboratory animal care. Better and fuller factual data on animal pain recognition and treatment will allow clearer focus on the ethical questions, which require attention both to fact and value. Good people can place different values on the need to avoid animal suffering, the need to promote medical progress, and where to place the benefit of the doubt when the facts are not entirely known or outcomes entirely predictable. Despite progress, a goal of *zero* unalleviated laboratory animal pain could be achieved in the near future only by deciding to abandon some types of animal studies.
